# 

**Published:** 1887-01-15

**Authors:** 


PRICE ONE PENNY.

No. 16, Vol. I.
January 15th, 1887.

Per Annum, 6s. 6d., post free.
Monthly Parts, 6d.; post free, 7½d.
Ditto per Ann., 7s. 6d., post free.

